# Supplementary material for: Fast Detection of Two Smenamide Family Members Using Molecular Networking
Source: Mar Drugs. 2019 Oct 30;17(11):618. doi: 10.3390/md17110618 (PMC6891588; doi:10.3390/md17110618)

**Supplementary Information**

**Table S1.** NMR data of smenamide F (**1**) (CD_3_OD).

**Table S2.** NMR data of smenamide G (**2**) (CD_3_OD).

**Figure S1.** ECD spectra of smenamide A, smenamide F (**1**) and G (**2**).

**Figure S2.** Positive ion mode high-resolution ESI mass spectrum of smenamide F (**1**).

**Figure S3.** Positive ion mode high-resolution ESI MS/MS spectrum of smenamide F (**1**).

**Figure S4.** ^1^H-NMR spectrum of smenamide F (**1**) (700 MHz, CD_3_OD).

**Figure S5.** COSY spectrum of smenamide F (**1**) (700 MHz, CD_3_OD).

**Figure S6.** NOESY spectrum of smenamide F (**1**) (700 MHz, CD_3_OD).

**Figure S7.** HSQC spectrum of smenamide F (**1**) (700 MHz, CD_3_OD).

**Figure S8.** HMBC spectrum of smenamide F (**1**) (700 MHz, CD_3_OD).

**Figure S9.** Positive ion mode high-resolution ESI mass spectrum of smenamide G (**2**).

**Figure S10.** Positive ion mode high-resolution ESI MS/MS spectrum of smenamide G (**2**).

**Figure S11.** ^1^H-NMR spectrum of smenamide G (**2**) (700 MHz, CD_3_OD).

**Figure S12.** COSY spectrum of smenamide G (**2**) (700 MHz, CD_3_OD).

**Figure S13.** ROESY spectrum of smenamide G (**1**) (700 MHz, CD_3_OD).

**Figure S14.** HSQC spectrum of smenamide G (**2**) (700 MHz, CD_3_OD).

**Figure S15.** HMBC spectrum of smenamide G (**2**) (700 MHz, CD_3_OD).

**Figure S16.** Real-time dynamic monitoring of osteosarcoma cancer cells (MG-63) proliferation after treatment with smenamides F (**1**) and G (**2**), using the RTCA platform.

**Table S1.** NMR data of smenamide F (**1**) (CD_3_OD).

| Position |  | δ_H_ [mult., *J* (Hz)] | δ_C_ [mult.] | δ_H_ [mult., *J* (Hz)] | δ_C_ [mult.] |
| --- | --- | --- | --- | --- | --- |
| 1 |  | - | 135.9 (C) | - | 135.9 (C) |
| 2/6 |  | 7.16 | 131.3 (CH) | 7.16 | 131.3 (CH) |
| 3/5 |  | 7.21 (m) | 129.3 (CH) | 7.21 | 129.3 (CH) |
| 4 |  | 7.18 | 128.1 (CH) | 7.18 | 128.1 (CH) |
| 7 | a | 3.53 (dd, 13.9, 4.5) | 35.8 (CH_2_) | 3.53 (dd, 13.9, 4.5) | 35.8 (CH_2_) |
|  | b | 3.11 (dd, 13.9, 3.4) |  | 3.11 (dd, 13.9, 3.4) |  |
| 8 |  | 4.90 (dd, 4.5, 3.4) | 61.5 (CH) | 4.90 (dd, 4.5, 3.4) | 61.5 (CH) |
| 9 |  | - | 180.4 (C) | - | 180.4 (C) |
| 10 |  | 4.90 (br. s) | 95.7 (CH) | 4.91 (br. s) | 95.7 (CH) |
| 11 |  | - | 172.5(C) | - | 172.5(C) |
| 12 |  | - | 178.1 (C) | - | 178.1 (C) |
| 13 |  | 4.08 (m) | 44.4 (CH) | 4.09 (m) | 44.4 (CH) |
| 14 |  | 1.02 (d, 6.9) | 14.7 (CH_3_) | 1.03 (d, 6.9) | 14.7 (CH_3_) |
| 15 |  | 3.99 (dd, 9.6, 2.5) | 75.8 (CH) | 3.98 (dd, 9.6, 2.4) | 75.7 (CH) |
| 16 |  | 1.66 (m) | 34.9 (CH) | 1.67 (m) | 34.9 (CH) |
| 17 |  | 0.94 (d, 7.0) | 12.7 (CH_3_) | 0.95 (d, 7.0) | 12.8 (CH_3_) |
| 18 | a | 1.64 | 33.4 (CH_2_) | 1.66 | 33.4 (CH_2_) |
|  | b | 1.45 |  | 1.46 |  |
| 19 |  | 2.21 | 33.4 (CH_2_) | 2.23 | 33.4 (CH_2_) |
| 20 |  | - | 143.7 (C) | - | 143.5 (C) |
| 21 |  | 5.97 (br. s) | 113.8 (CH) | 6.02 (br. s) | 114.2 (CH) |
| 22 |  | 2.25 | 28.2 (CH_2_) | 2.28 | 28.0 (CH_2_) |
| 23 |  | 1.71 | 25.8 (CH_2_) | 1.78 | 26.8 (CH_2_) |
| 24 |  | 3.39 | 48.6 (CH_2_) | 3.39 | 51.6 (CH_2_) |
| 25 |  | - | 173.4 (C) | - | 173.2 (C) |
| 26 |  | 2.09 (s) | 21.8 (CH_3_) | 2.11 (s) | 21.2 (CH_3_) |
| 27 |  | 3.07 (s) | 36.7 (CH_3_) | 2.93 (s) | 33.7 (CH_3_) |
| OMe |  | 3.82 (s) | 59.4 (CH_3_) | 3.83 (s) | 59.4 (CH_3_) |

**Table S2.** NMR data of smenamide G (**2**) (CD_3_OD).

|  |  | Conformer 1 (major) | | Conformer 2 (minor) | |
| --- | --- | --- | --- | --- | --- |
| Position |  | δ_H_ [mult., *J* (Hz)] | δ_C_ [mult.] | δ_H_ [mult., *J* (Hz)] | δ_C_ [mult.] |
| 1 |  | - | 135.4 (C) | - | 135.4 (C) |
| 2/6 |  | 6.95 (dd, 7.8, 2.1) | 131.0 (CH) | 6.95 (dd, 7.8, 2.1) | 131.0 (CH) |
| 3/5 |  | 7.21 | 129.2 (CH) | 7.21 | 129.2 (CH) |
| 4 |  | 7.20 | 128.2 (CH) | 7.20 | 128.2 (CH) |
| 7 | a | 3.57 (dd, 13.9, 5.3) | 35.6 (CH_2_) | 3.57 (dd, 13.9, 5.3) | 35.6 (CH_2_) |
|  | b | 3.14 (dd, 13.9, 2.6) |  | 3.14 (dd, 13.9, 2.6) |  |
| 8 |  | 4.90 (sovr. acqua) | 61.1 (CH) | 4.90 (sovr. acqua) | 61.1 (CH) |
| 9 |  | - | 180.0 (C) | - | 180.0 (C) |
| 10 |  | 5.02 (br. s) | 96.0 (CH) | 5.02 (br. s) | 96.0 (CH) |
| 11 |  | - | 172.1(C) | - | 172.1(C) |
| 12 |  | - | 177.7 (C) | - | 177.7 (C) |
| 13 |  | 4.08 (dq, 9.3, 7.0) | 43.8 (CH) | 4.09 (dq, 9.3, 7.0) | 43.8 (CH) |
| 14 |  | 1.06 (d, 7.0) | 15.1 (CH_3_) | 1.06 (d, 7.0) | 15.1 (CH_3_) |
| 15 |  | 3.86 (dd, 9.3, 2.1) | 75.9 (CH) | 3.86 (dd, 9.3, 2.1) | 75.9 (CH) |
| 16 |  | 1.61 (m) | 35.0 (CH) | 1.63 (m) | 35.0 (CH) |
| 17 |  | 0.87 (d, 6.8) | 12.9 (CH_3_) | 0.88 (d, 6.8) | 12.9 (CH_3_) |
| 18 | a | 1.56 | 33.2 (CH_2_) | 1.57 | 33.2 (CH_2_) |
|  | b | 1.38 |  | 1.40 |  |
| 19 |  | 2.19 | 33.3 (CH_2_) | 2.20 | 33.3 (CH_2_) |
| 20 |  | - | 143.4 (C) | - | 143.3 (C) |
| 21 |  | 5.96 (br. s) | 113.8 (CH) | 6.00 (br. s) | 114.2 (CH) |
| 22 |  | 2.23 | 28.2 (CH_2_) | 2.27 | 28.0 (CH_2_) |
| 23 |  | 1.69 | 25.8 (CH_2_) | 1.77 | 26.8 (CH_2_) |
| 24 |  | 3.39 | 48.6 (CH_2_) | 3.40 | 51.6 (CH_2_) |
| 25 |  | - | 173.3 (C) | - | 173.1 (C) |
| 26 |  | 2.09 (s) | 21.8 (CH_3_) | 2.11 (s) | 21.2 (CH_3_) |
| 27 |  | 3.07 (s) | 36.7 (CH_3_) | 2.93 (s) | 33.7 (CH_3_) |
| OMe |  | 3.93 (s) | 59.5 (CH_3_) | 3.93 (s) | 59.5 (CH_3_) |

**Figure S1.** ECD spectra of smenamide A, smenamide F (**1**) and G (**2**).

**
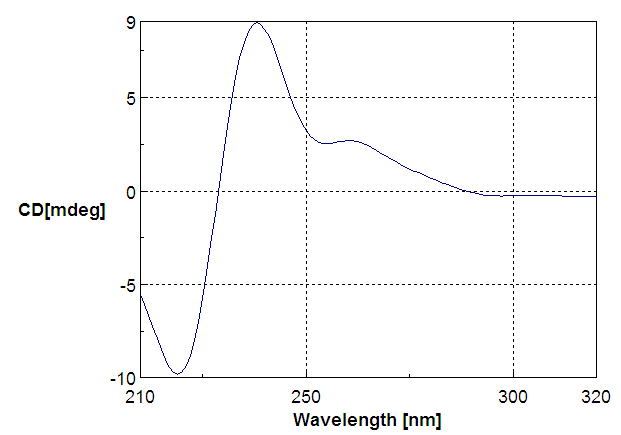

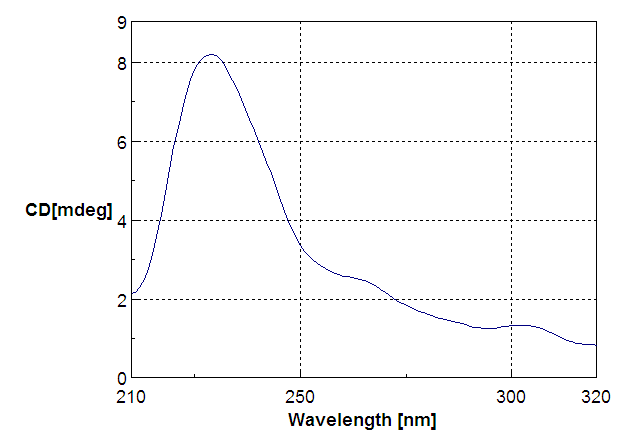
**

**smenamide A smenamide F**

**
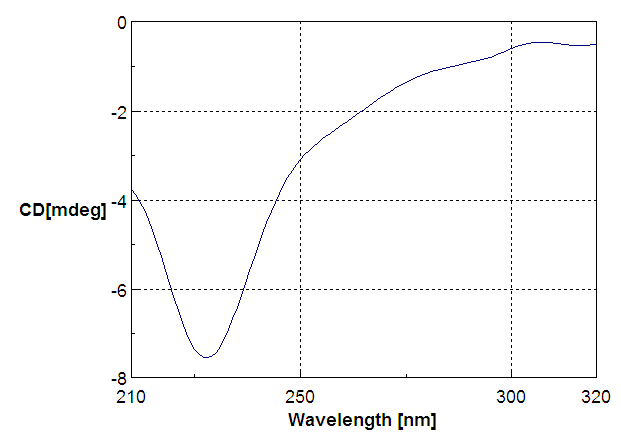
**

**smenamide G**

**Figure S2.** Positive ion mode high-resolution ESI mass spectrum of smenamide F (**1**).

**Figure S3.** Positive ion mode high-resolution ESI MS/MS spectrum of smenamide F (**1**)

**
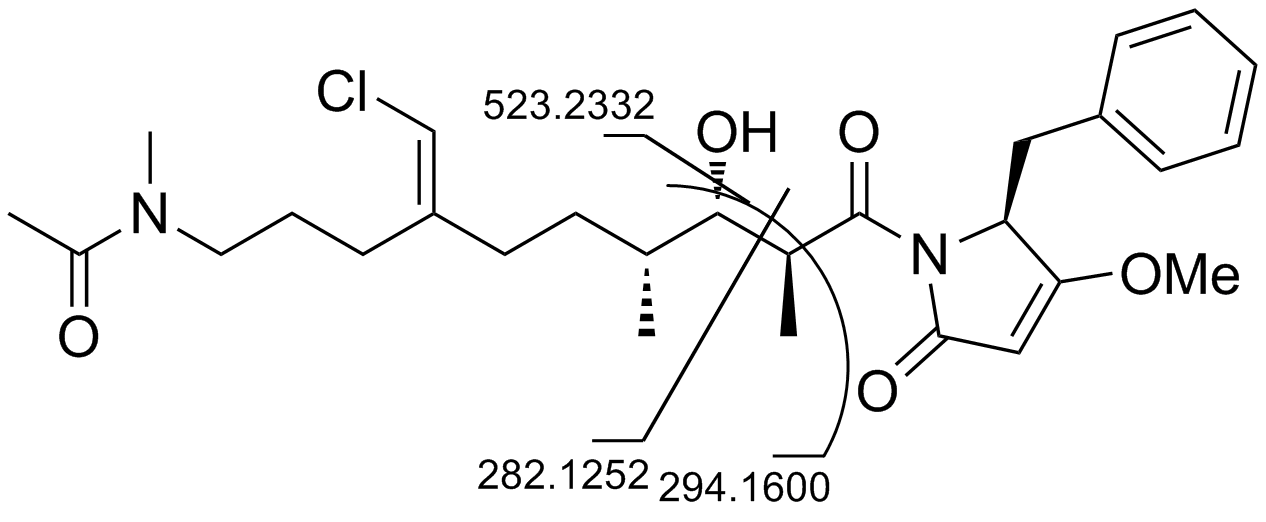
**

**Figure S4.** ^1^H-NMR spectrum of smenamide F (**1**) (700 MHz, CD_3_OD).


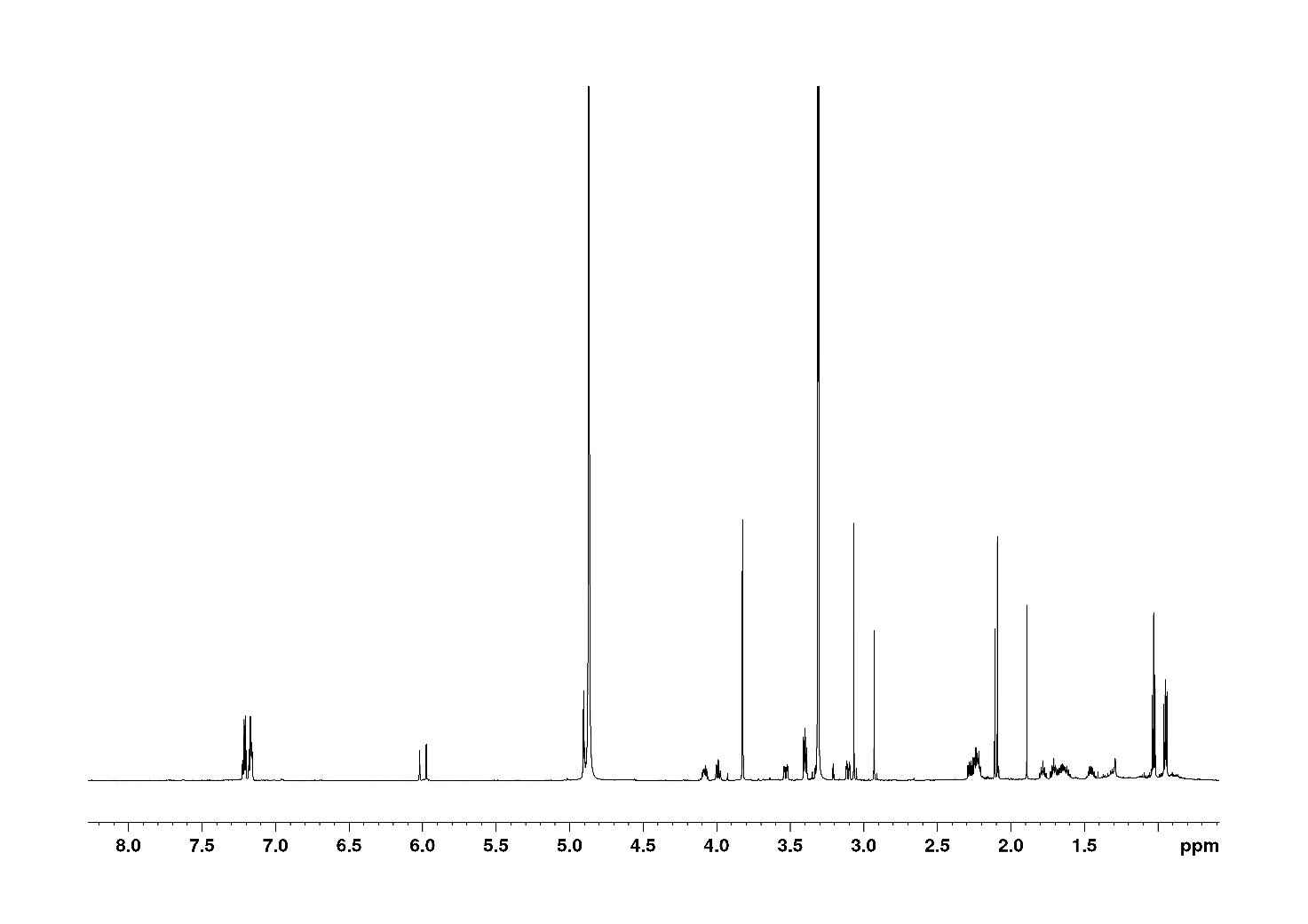


**Figure S5.** COSY spectrum of smenamide F (**1**) (700 MHz, CD_3_OD).


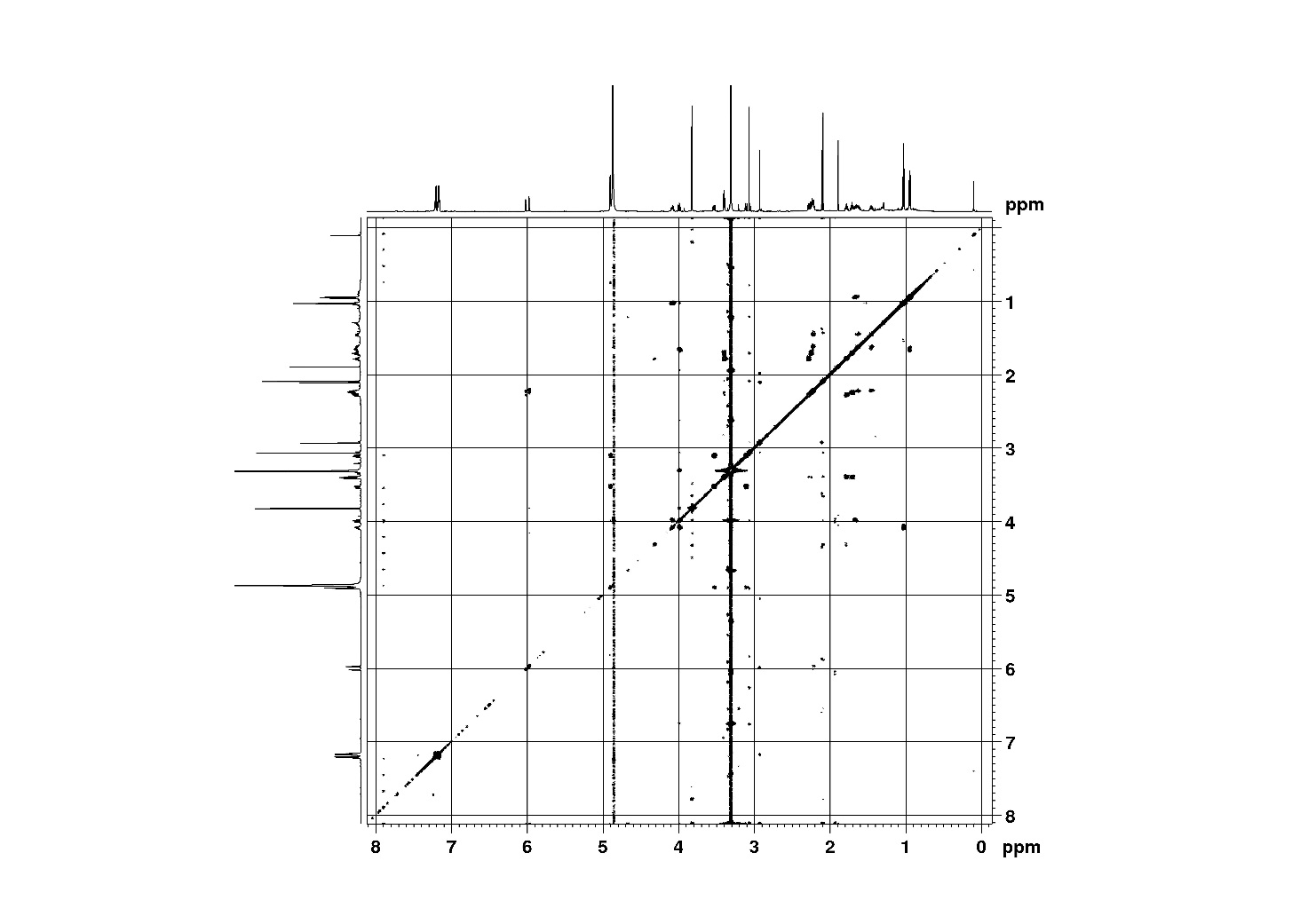


**Figure S6.** NOESY spectrum of smenamide F (**1**) (700 MHz, CD_3_OD).


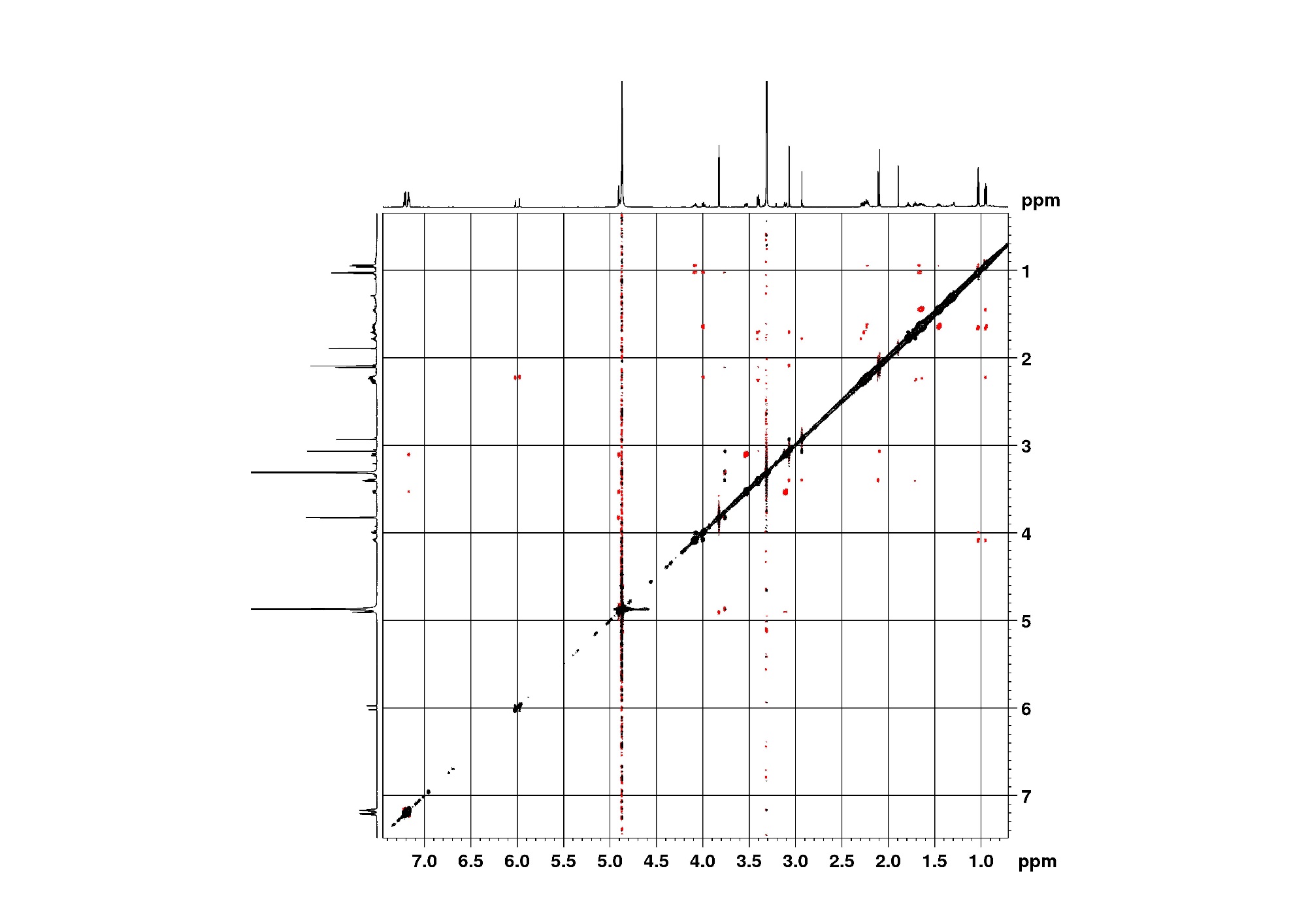


**Figure S7.** HSQC spectrum of smenamide F (**1**) (700 MHz, CD_3_OD).


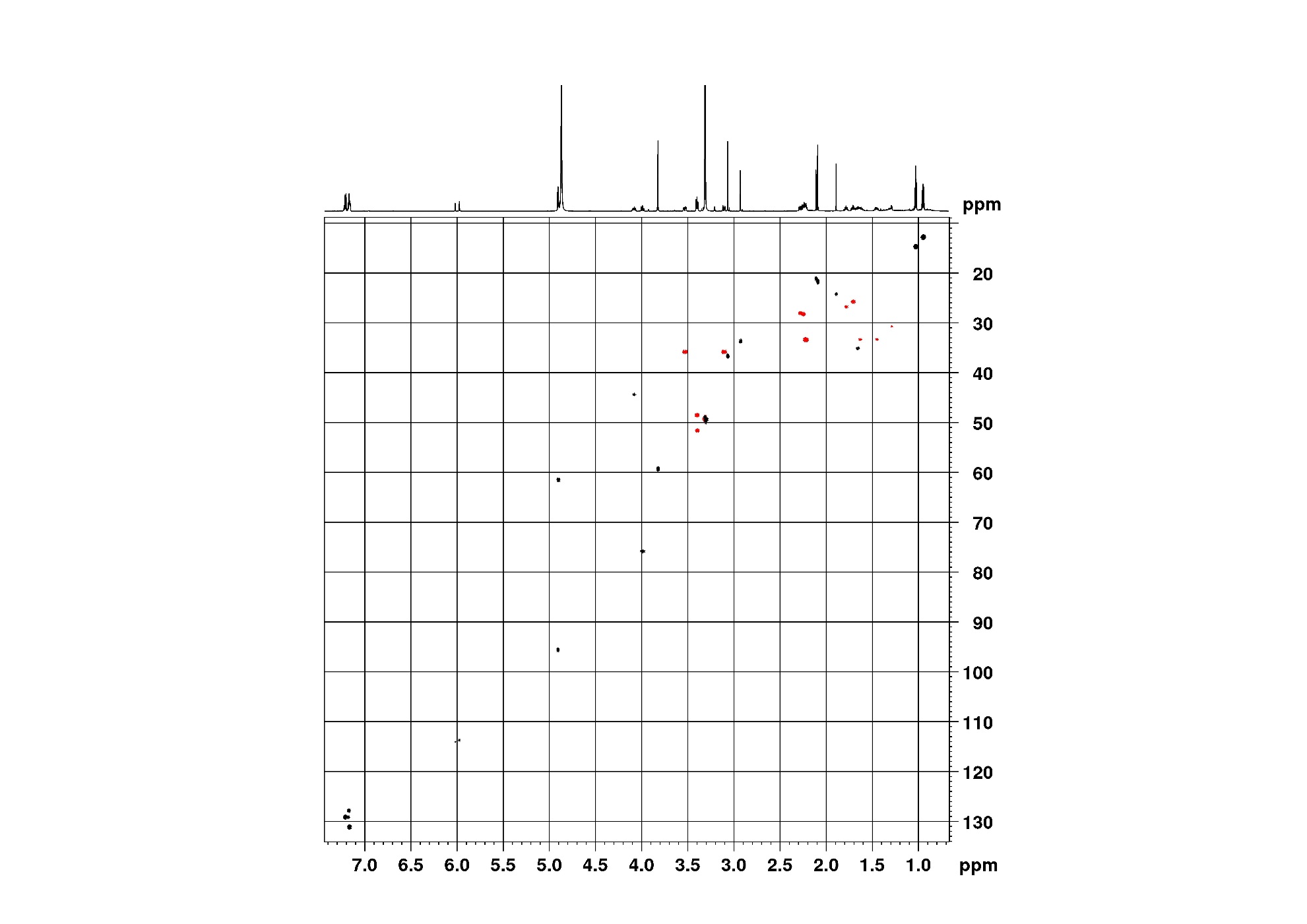


**Figure S8.** HMBC spectrum of smenamide F (**1**) (700 MHz, CD_3_OD).


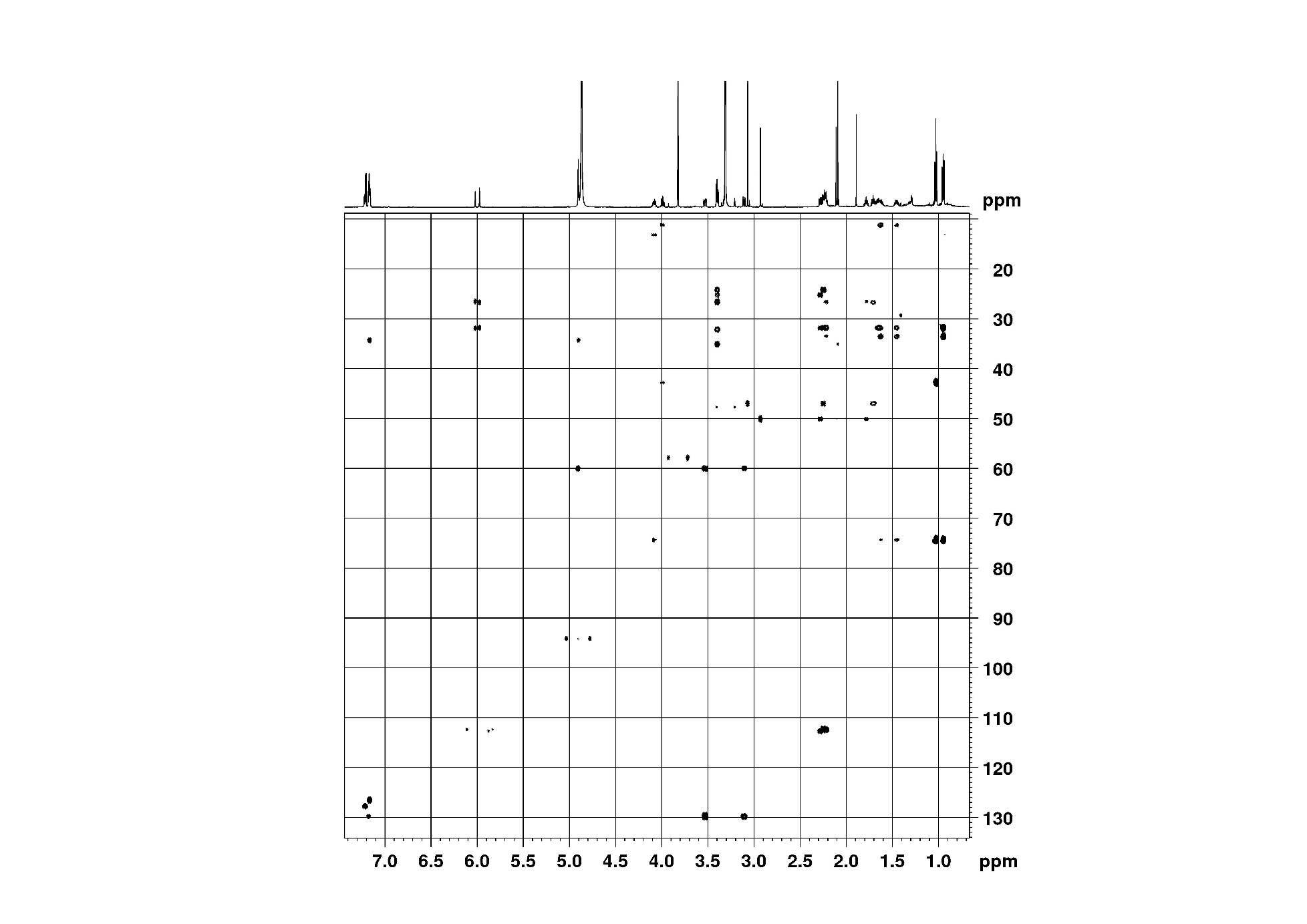


**Figure S9.** Positive ion mode high-resolution ESI mass spectrum of smenamide G (**2**).

**Figure S10.** Positive ion mode high-resolution ESI MS/MS spectrum of smenamide G (**2**).

**
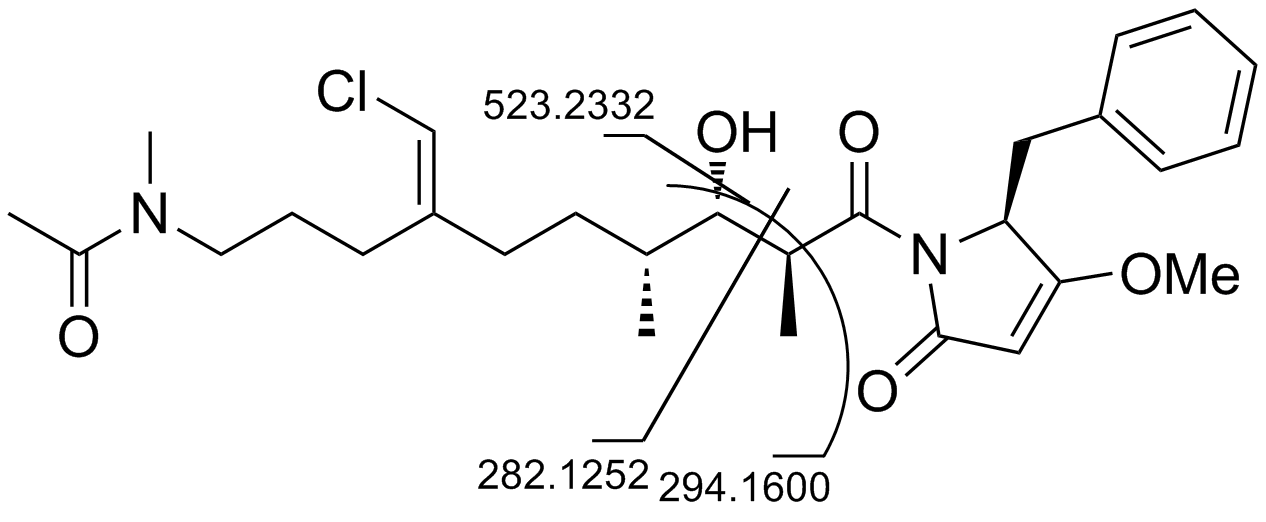
**

**Figure S11.** ^1^H-NMR spectrum of smenamide G (**2**) (700 MHz, CD_3_OD).


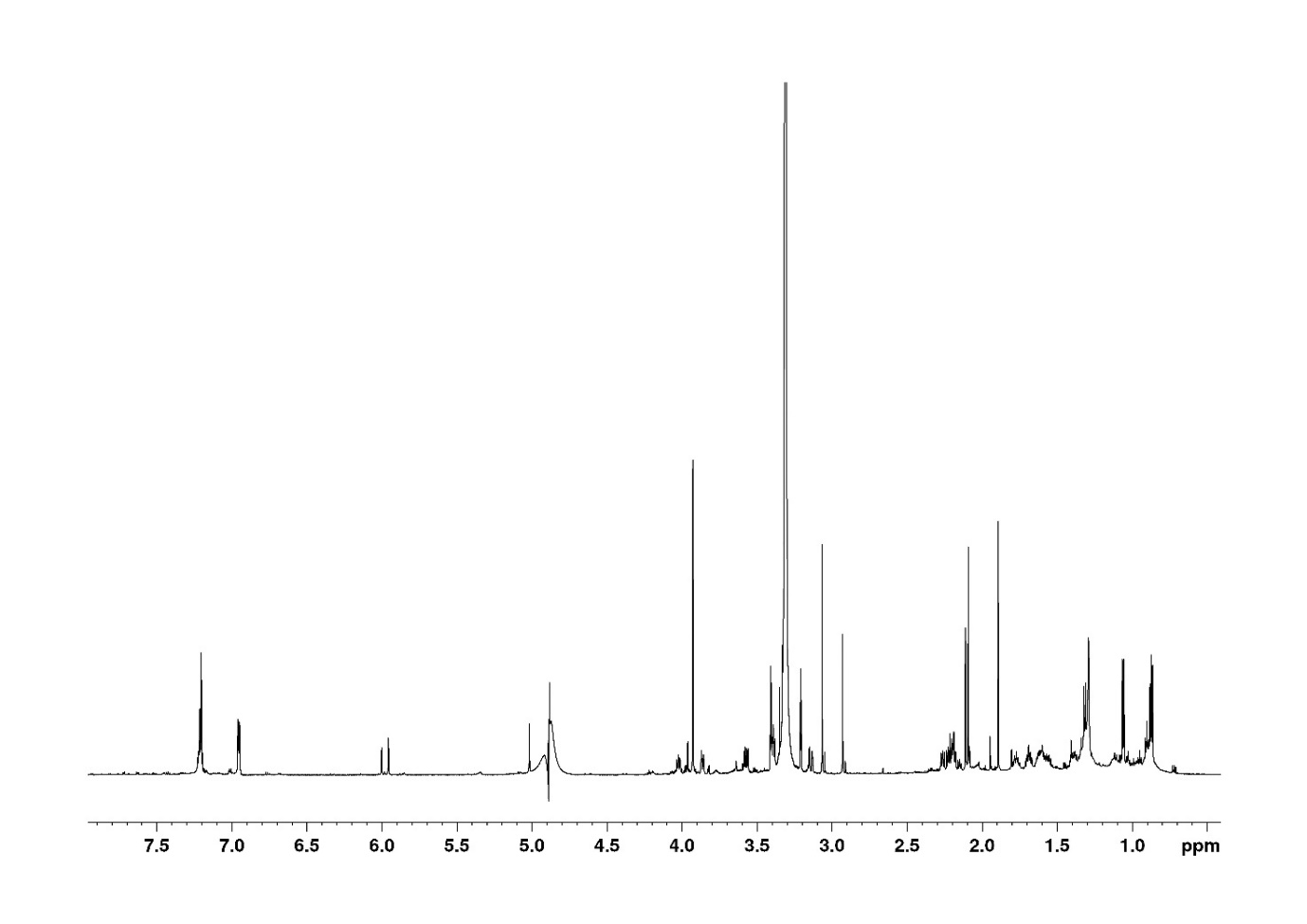


**Figure S12.** COSY spectrum of smenamide G (**2**) (700 MHz, CD_3_OD).


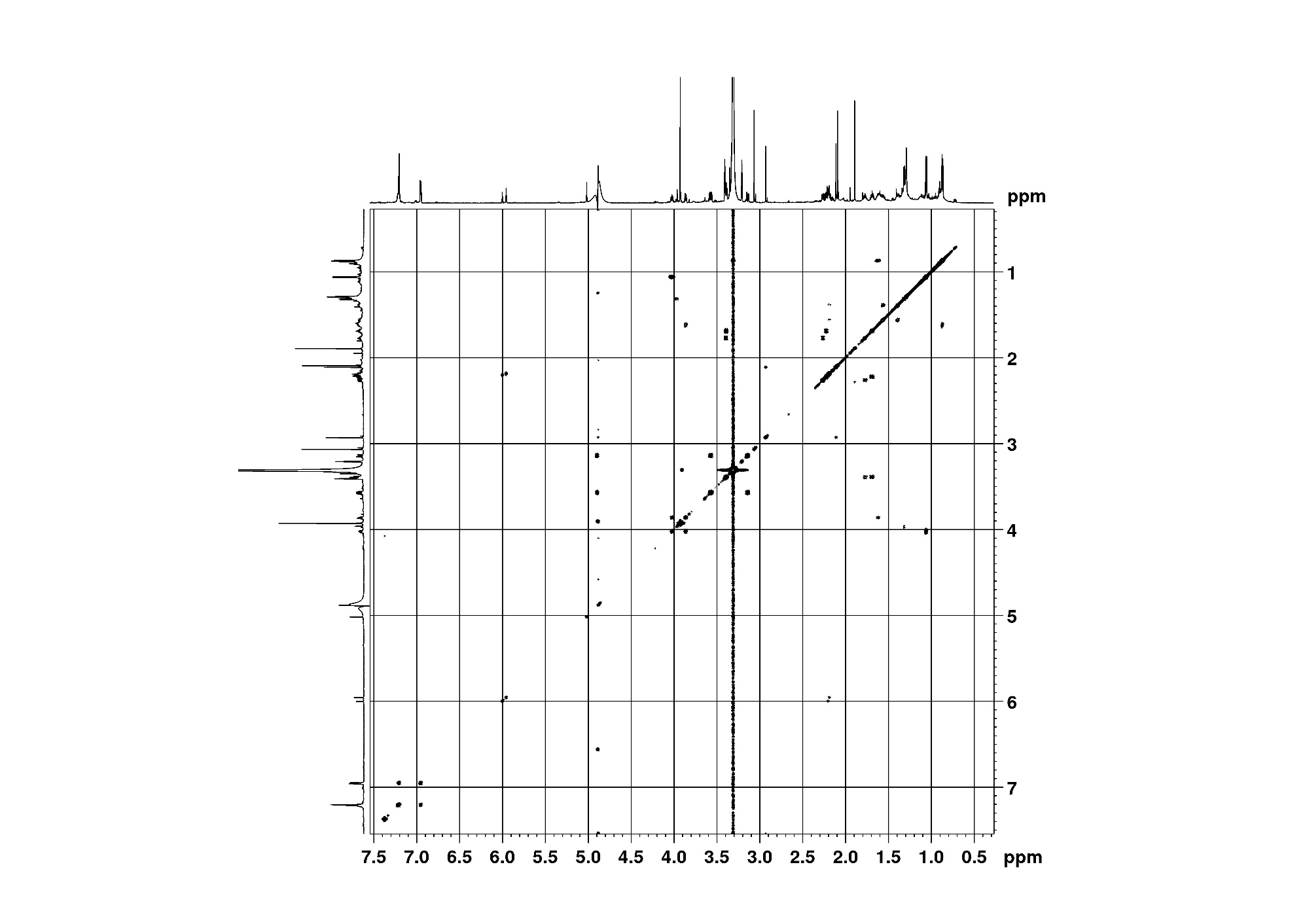


**Figure S13.** ROESY spectrum of smenamide G (**2**) (700 MHz, CD3OD).


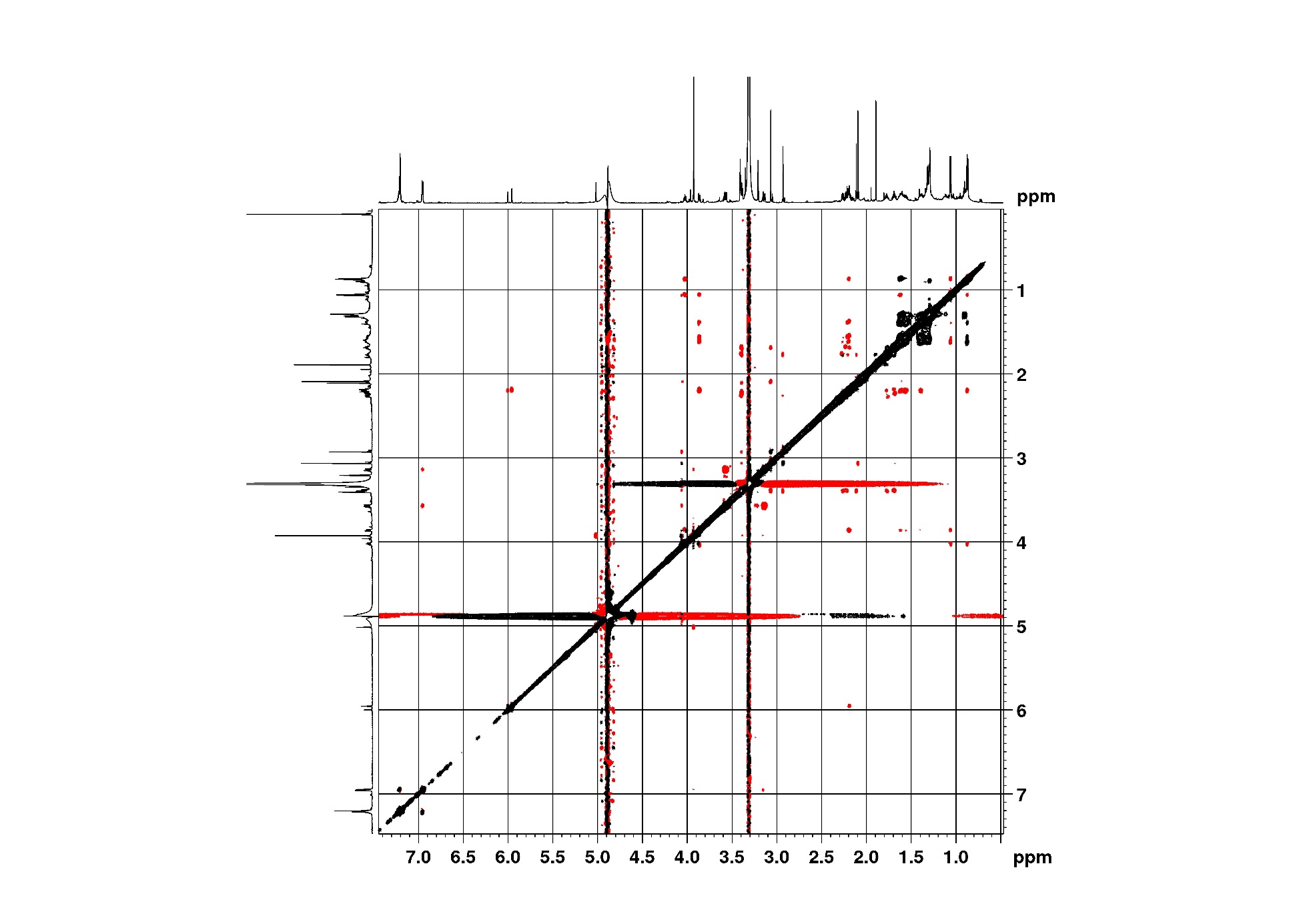


**Figure S14.** HSQC spectrum of smenamide G (**2**) (700 MHz, CD_3_OD).


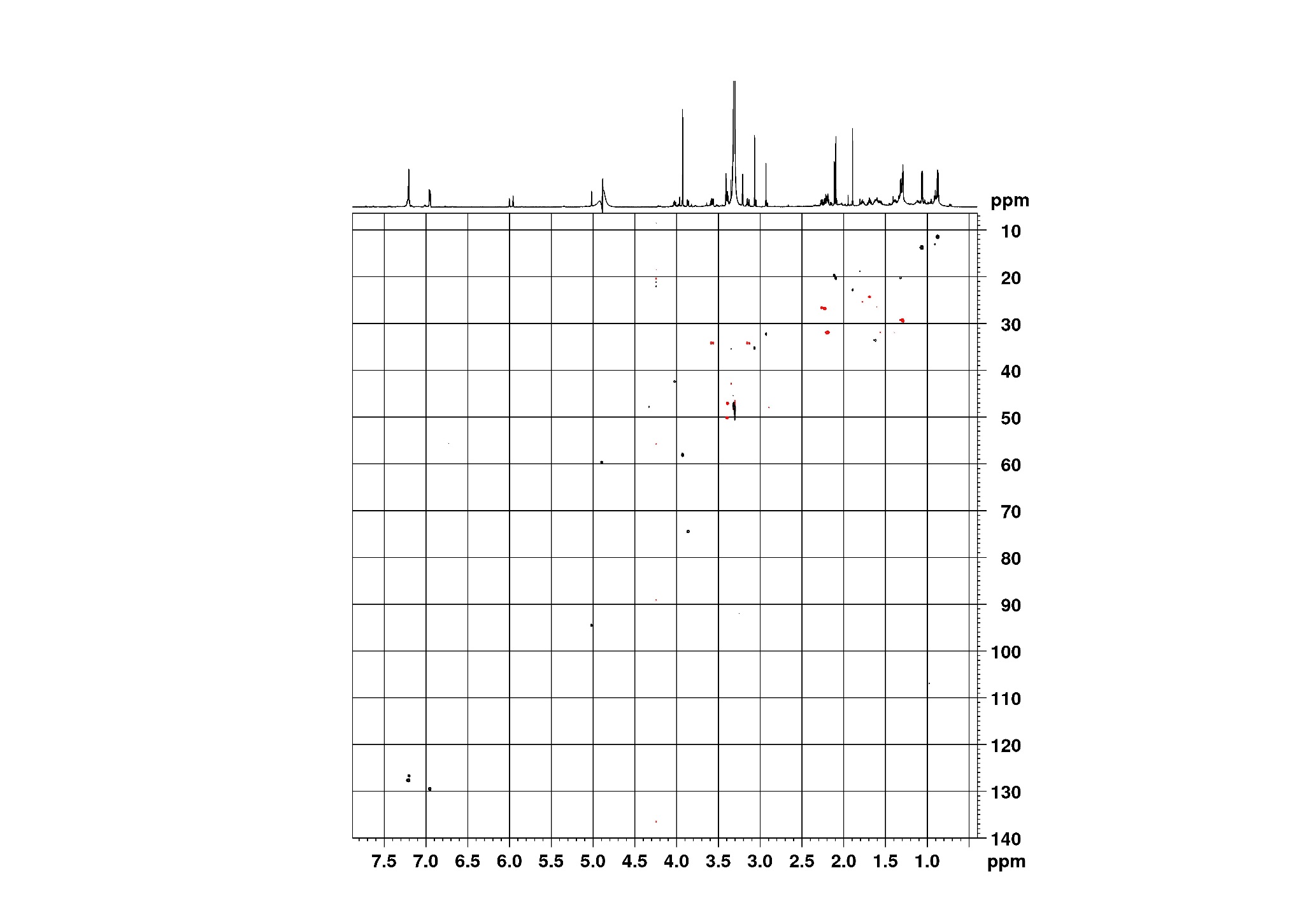


**Figure S15.** HMBC spectrum of smenamide G (**2**) (700 MHz, CD_3_OD).


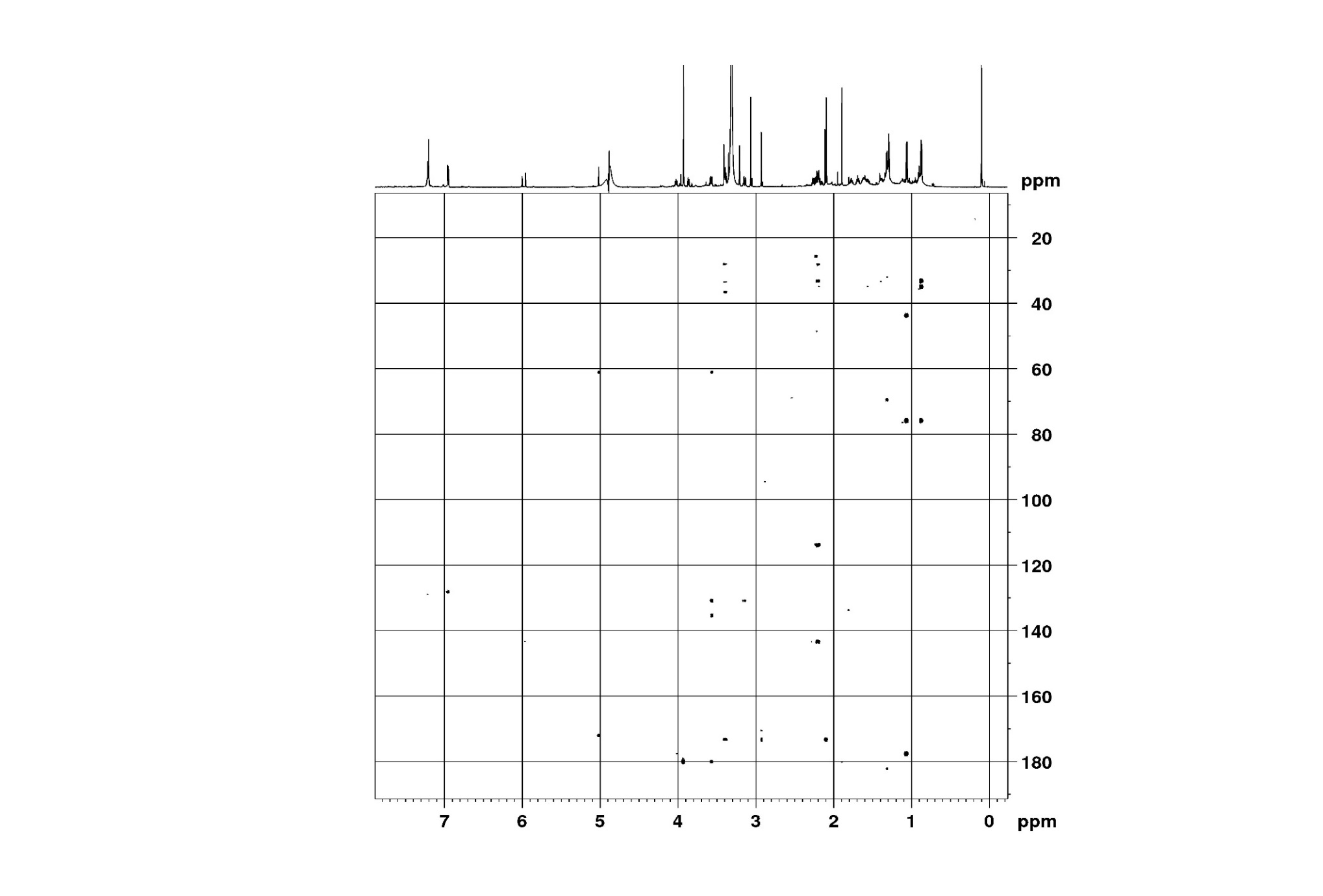


**Figure S16**. Real-time dynamic monitoring of osteosarcoma cancer cells (MG-63) proliferation after treatment with smenamides F and G, using the RTCA platform. (A) Normalized cell index (NCI) kinetics of MG-63 cells after exposure to 5 μM of smenamides F and G, and to 0.5 % DMSO vehicle control for 96 hours. Black arrow shows the starting point of drug treatment. Each cell index value was normalized just before treatment. (B) NCI variations of MG-63 cells after 96h exposure to 5 μM of smenamides F and G, and to 0.5 % DMSO vehicle control. Antiproliferative effects are reported as slope of NCI to describe the changing rate of growth curves after drug treatment. NCI slope values are relative to controls treated with DMSO vehicle. (C) Doubling times of NCI of MG-63 after exposure to 5 μM of smenamides F and G, and 0.5% DMSO. Data are presented as mean ± SD; n=3.


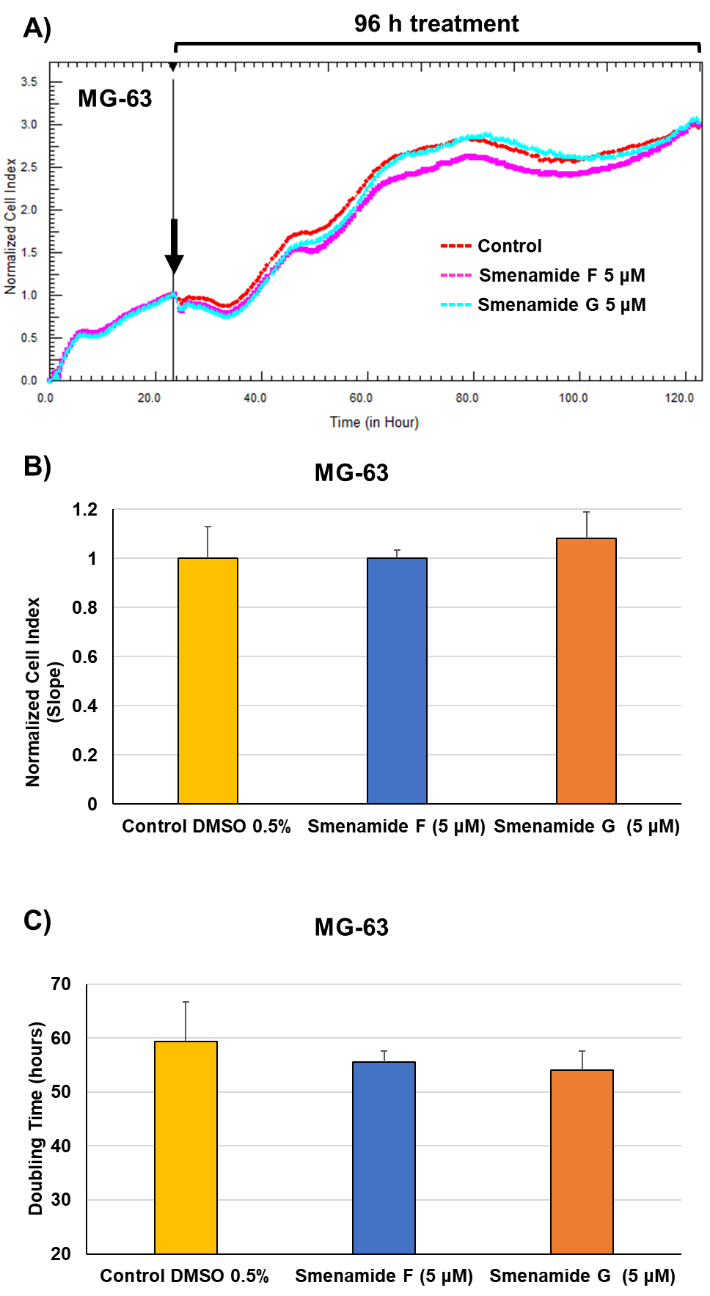

Supplement: Supplementary file 1 [file marinedrugs-17-00618-s001.zip › smenamides F and G_Supplementary material.docx]
